# Process epistemology in the COVID-19 era: rethinking the research process to avoid dangerous forms of reification

**DOI:** 10.1007/s13194-022-00450-4

**Published:** 2022-03-07

**Authors:** John Dupré, Sabina Leonelli

**Affiliations:** 1 Exeter Centre for the Study of the Life Sciences (Egenis), University of Exeter, Exeter, UK; 2 Wissenschaftskolleg zu Berlin, Wallotstrasse 19, 14193 Berlin, Germany

**Keywords:** Scientific practice, Biology, Models, Data, Knowledge, Pandemic

## Abstract

Whether we live in a world of autonomous things, or a world of interconnected processes in constant flux, is an ancient philosophical debate. Modern biology provides decisive reasons for embracing the latter view. How does one understand the practices and outputs of science in such a dynamic, ever-changing world - and particularly in an emergency situation such as the COVID-19 pandemic, where scientific knowledge has been regarded as bedrock for decisive social interventions? We argue that key to answering this question is to consider the role of the activity of *reification* within the research process. Reification consists in the identification of more or less stable features of the flux, and treating these as constituting stable things. As we illustrate with reference to biological and biomedical research on COVID-19, reification is a necessary component of any process of inquiry and comes in at least two forms: (1) means reification (phenomena-to-object), when researchers create objects meant to capture features of the world, or phenomena, in order to be able to study them; and (2) target reification (object-to-phenomena), when researchers infer an understanding of phenomena from an investigation of the epistemic objects created to study them. We note that *both* objects and phenomena are dynamic processes and argue that have no reason to assume that changes in objects and phenomena track one another. We conclude that failure to acknowledge these forms of reification and their epistemic role in scientific inquiry can have dire consequences for how the resulting knowledge is interpreted and used.

## Introduction

A few decades ago it was widely believed that the core problems in the philosophy of science were problems of language. This seems strange to many today. But it is not hard to understand the reasoning. Philosophers knew, or thought they knew, that there were kinds of things out there with specific structures and properties, things that science aimed to identify and name. Following Frege, it was supposed that a word was connected to the things it named by beliefs about those things that constituted the sense, or meaning, of the word. But this raised a problem that as scientific theories developed or were replaced, our beliefs about the entities science described changed, thus, presumably, changing the meanings of the words referring to them. And if words, or at least their meanings, were fluid, how could they successfully refer to the unchanging kinds of things in the world? The celebrated solution to this problem was provided by Putnam and Kripke. The meanings of scientific words, ‘natural kind terms’, did not really change. What mattered was their reference, and this remained fixed once an exemplar of the kind had been dubbed with a scientific term.

The idea that there is, or can be, a fixed link between a scientific concept and a referent in the world continues to have mileage in contemporary philosophy. The problem that we want to explore in this paper is that neither of the relata of this supposed link are in fact fixed. As process ontologists have recently insisted, the targets of biological investigation, far from being fixed are constantly metabolising, developing, and evolving (Dupré, 2021). They are not things at all, but processes. And indeed, it is possible to extend this view even to the non-living world, thereby providing a compelling case for adopting a general process ontology (Dupré & Nicholson, 2018). Meanwhile the ways that science connects with these targets of investigations and attempts to capture them, now expanded to include not just concepts, but models, data and more, are themselves fluid, developing through scientific activity and changes in scientific context (Rheinberger, 1997, Soler et al., 2014, Leonelli, 2016). So it appears that the Putnam/Kripke move was the opposite of the correct one. Far from finding a fixed phenomenon to stabilise the concept, the growth of scientific knowledge has destabilised the epistemic object by creating a wealth of ever-shifting, inter-dependent methods and tools with which to interact with the world.^1^

While a broader historical view of epistemic objects shows them to be fluid and changing, this is not typically how they are seen in the day-to-day life of the working scientist. To understand and intervene in the world, scientists produce models, data and theories that capture how the world is at a specific place and moment in time, and thus the parameters and criteria they use to represent the world are inherently static (even, as we shall see, when such parameters are specifically geared to capturing dynamic systems, as in the case of adaptive modelling and simulations). And this raises a problem that has not been adequately addressed within contemporary philosophy of science. With both an epistemic object that, in the longer time scale, is fluid, and a fluid subject of investigation, there is nothing to guarantee the stability of our scientific findings. It would seem like a cosmic coincidence if the changes to our scientific representations (a central class of epistemic objects) perfectly tracked the changes to the phenomena we sought to understand. In this paper we explore this problem and consider what approaches to scientific methodology might best mitigate it. We thus ask, how do we understand the stabilising practices and stabilised outputs of science as tools to explore a dynamic, ever-changing world? Are scientific outputs reliable means of understanding processes, and what are the practical and conceptual implications of adopting research methods that systematically constrain or even disregard the dynamic aspects of phenomena? Does any attempt to quantify, explain, or intervene involve an implicit rejection of process ontology, and if so, does that weaken the credibility of this ontological view or, on the other hand, of science as a system of knowledge production?

To illustrate and exemplify the significance of the problem and its implications for both philosophy and the sciences, in this paper we consider these questions with reference to the emergency situation created by the coronavirus pandemic, in which the tension between the dynamic, rapidly changing nature of biological and social reality and the essentialising tendencies entrenched in biomedical knowledge production have become readily apparent. On the one hand, scientific knowledge of COVID-19 played a crucial role in informing public responses to the pandemic, including radical forms of social intervention. Its authoritative status came from trust in the empirical nature of such knowledge, confidence that it is grounded on hard-won evidence about how the world works, including reliable data and models that accurately represent the phenomena at hand. On the other hand, the agent at the heart of the pandemic – the SARS-CoV-2 virus – is among the least stable of evolving forms, highly responsive to the ever-changing features of its organic and inorganic environments and likely to change rapidly as a result. Any attempt to stabilise viral infections enough to be able to study them requires procedures that we call “reification”, consisting in the identification and treatment of relevant features of the world as stable things for the purposes of inquiry, thus disregarding – at least temporarily – their volatile, changeable nature.^2^ While researchers study reified versions of the virus, its real-world environments change very rapidly and newly evolved variants of the virus continue to emerge, resulting in grave concern that the effectiveness of vaccines devised to address the pandemic may be threatened by mutated variants. As we shall argue, this is a case where failure to question reification leads to problematic uses of models and data, thus threatening the very foundations of the empiricist trust in the reliability of scientific methods.

We argue that while reification is a necessary component of any process of inquiry, there are significant consequent risks associated with producing scientific knowledge; and that such risks can be mitigated by explicitly acknowledging the forms of reification involved in research and their potential implications for interpreting scientific findings – in ways that COVID biomedicine, in its haste to inform the pandemic response, has sometimes failed to do. In other words, we use examples from COVID research to argue that reification is particularly pernicious when implicit confusions between momentary snapshots of processes and unchanging things become entrenched in how we understand the world and thereby hard to identify and problematize, even as they prove damaging in their scientific and social implications. This is not a novel philosophical position, with philosophers ranging from Karl Marx to John Dewey pointing to the epistemic dangers of reification.^3^ Essentially the same problem is memorably referred to by Alfred North Whitehead as ‘the fallacy of misplaced concreteness’ (Whitehead, 1929, 11). Our aim in this paper is to update those arguments to embrace contemporary insights from process ontology and the philosophy of science, and to point to their implications for both philosophical and scientific approaches to empirical research, including the interpretation of emerging knowledge on COVID and its likely transmission pattern. In particular, we aim to defend a processual approach to scientific epistemology which highlights the dynamic relation between the objects produced through research practices and the phenomena that these objects are used to study, and we argue for the usefulness of this approach in framing scientific inquiry and interpreting its results.

The paper begins with a brief introduction to the process view of ontology, focusing on biology and then more specifically on viruses. We then turn to the idea of a process epistemology and distinguish between two stages of reification that are associated with scientific inquiry: (1) means-reification (phenomenon-to-object), when researchers create objects such as data and models that are meant to capture features of the world at a particular point in time, or phenomena, in order to be able to study them; and (2) target-reification (object-to-phenomenon), when researchers infer a understanding of features of the world from an understanding of the objects created to study them. We then illustrate how these forms of reification have worked in biological and biomedical research on COVID-19, emphasising that the objects that scientists create are not only static approximations to dynamic processes in the world but also abstractions from the process or flow of scientific activity. Both these underlying processes may, as COVID research demonstrates, undergo secular change as well as the regular, often cyclical, activities that sustain them, and attempting to align changes in the phenomena with changes to scientific objects is a constant and not always successful struggle. Starting from examples drawn from this research area, we reflect on (1) the opportunities created by scientific approaches that explicitly problematize reification, and its implications for prospective uses of the knowledge being produced, and (2) the risks involved in failing to acknowledge the existence of reification and its epistemic role in scientific inquiry. We propose a process epistemology as a corrective to epistemic views which fail to stress the importance of remaining alert to reification and its consequences. We conclude with some brief reflections on the implications of this analysis for the general understanding of knowledge and its multiple contexts.

## Process ontology: The case of biology

Two contrasting visions of the world have resonated through Western philosophy since the pre-Socratics. On one side is the world of things or substances, distinct beings defined by unchanging, essential properties, in their purest form the immortal and immutable atoms of philosophers such as Leucippus and Democritus. On the other side is the world of constant change, or process, imagined by Heraclitus, a world in which everything flows, and nothing stays the same from moment to moment. A starting point for this essay is that a proper understanding of contemporary biology – and arguably, though we cannot make the argument here, the world more generally - provides an irresistible push towards the second of these visions.^4^

Here we can do no more than summarise the grounds for this view of biology, by sketching three reasons why an organism, a central biological category if anything is, should be seen as a process rather than a thing. Similar arguments could be provided for other key biological entities.^5^ First, contrary to the idea that a thing is by default stable and self-sufficient, an organism is a system in severe thermodynamic disequilibrium, which maintains its integrity only by a complex hierarchy of metabolic activities, fuelled by energy captured from the environment. Second, the persistence of a thing is generally taken to depend on some constant, perhaps essential, properties. But the developmental trajectory of an organism through stages such as egg, larva, pupa and adult in an insect, displays no such constant properties.^6^ The stages are connected by causal links rather than constant properties. And finally, contradicting again the self-sufficiency of a thing, most organisms are deeply and necessarily symbiotic. The intimacy of the relations between symbionts, for example between a multicellular eukaryote and its symbiotic microbes, is such as to threaten one final aspect of traditional thing thinking, the assumption of reasonably sharp boundaries. Whether the fungus and the photosynthetic bacteria in a lichen are distinct organisms in close contact, or parts of a multispecies organism, is not a question that admits of any objective resolution.

Thus, in summary, an oak tree or a horse have seemed to many philosophers to be paradigm things. Yet on closer examination they prove to be structures constantly maintained by countless internal activities and external interactions. Their clear boundaries turn out to be illusory when the trillions of symbiotic organisms are identified, permeating the roots and even connecting the tree through a mycelial web to other plants in its vicinity for the sharing of nutrients. And no one would suppose without observing the connecting process that the acorn was the same organism, or even the same kind of organism, as the mighty oak tree. These wider and deeper views of the tree are far better accommodated by the concept of a process, an almost unimaginably complex eddy in a sea of interacting processes that stabilise the structure of the organism (homeostasis) and also its developmental trajectory through time (homeorhesis).

Contrary to appearances, then, the individuality of a tree is not something given by default as long as nothing too drastic (fire, chain saws) intervenes, but a remarkable achievement. Rather than processes being secondary to, and dependent, on things, it turns out that many putative things, organisms at least, owe their thing-like appearance to countless stabilising processes. One might call this extraordinary achievement reification, except that we shall have other uses for that word in what follows. As noted above, we will use the term “reification” to refer to the stabilisation processes described above as parts of research practice, distinguishing between means-reification, which produces the tools and methods with which researchers interact with the world, and target-reification, the identification of specific aspects of the world about which researchers are producing knowledge. Crucially, we must not assume that the targets reified by scientists through the use of epistemic objects are identical to the processes, such as organisms, stabilised by nature.

## Process epistemology: Focusing on reification

Our aim in this paper is to ask how science interacts with such a processual world to generate knowledge, and how answers to this question may inform contemporary assessments of the reliability and trustworthiness of different kinds of research practice. That science itself is a process is not controversial (Hull, 1988). But how the process of science engages with a processual world raises questions which have not been fully appreciated. Science has often been understood as exploring the natures of fixed, perhaps eternally fixed, objects, even as disclosing their essences (Devitt, 2008; Ellis, 2001). Following the impetus provided by historical epistemology (Daston, 1994; Rheinberger, 1997) and the philosophy of science in practice (Hacking, 2002; Soler et al., 2014), we need to move away from such outmoded perspectives and ask how we should understand the practices and outputs of science in a dynamic, ever-changing world. We need a processual epistemology that properly complements a process-filled world.

We propose that the key to a processual epistemology is understanding what we shall call reification. Just as processes within the flux of living activity stabilise entities such as organisms and cells, so the activity of science stabilises entities in the flux of research such as data, models, classificatory labels (kinds) and explanatory concepts, all of which are used to capture aspects of the world in order to study them.^7^ We call these entities epistemic objects.^8^ Crucially, we cannot assume that the epistemic objects created by scientists correspond precisely to the entities stabilised by natural processes. A process epistemology starts from three well-trodden philosophical ideas that support this position, and which apply to all epistemic objects despite the widely different entities that we have garnered under that category. The first is the acknowledgment that – as extensively argued by philosophers working on scientific representation and experimentation – key research components such as models seldom consist of accurate representations of the world. Rather, models are often patently false representations, bearing little resemblance to their targets and yet fruitfully used to convey important insights about them.^9^ Moreover, models are inherently static at least in the sense that, no matter how successful models are in capturing the dynamic nature of specific targets, the parameters on which they are grounded are fixed and unable to capture shifts in the world without an iterative process of bootstrapping, whereby researchers revise and update the parameters operationalised in the model. This is the case even for machine learning systems, where parameters may automatically update on the basis of incoming information, but the rules according to which such updates happen are constrained by the initial programming and the characteristics of the data used to train the system. In the model, unlike in nature, there is always some level at which the rules are fixed.

The second well-trodden idea is an insistence on the unavoidably plural nature of research: there are many different processes through which both science (among other human activities) and nature create more or less stable “things”, and the resulting objects may, therefore, very well be highly diverse, hard to compare with each other and difficult to relate back to the processes that they are meant to document (Dupré, 1993, 2018). Data provide a striking example. Far from being simple “documents” of reality – “given”, as suggested by the Latin etymology of the term – data are the result of mediated and situated interactions between researchers and the world. Hence the characteristics of data, including their format, shape and content, depend *both* on the target system that researchers are attempting to document *and* on the instruments, methods and research environments through which data are generated. Even when investigating the same phenomenon, different research groups tend to produce diverse types of data, and comparing and integrating such different collections of epistemic objects often involves considerable effort. Data integration gets even harder when the data in question have been collected to study different phenomena in the first place (Leonelli & Tempini, 2018).

Third, and perhaps less widely appreciated, process epistemology involves an acknowledgment that research outputs are themselves processes which are only temporarily stabilised. Indeed, difficulties in interpreting and comparing the epistemic objects reified by researchers to study specific aspects of the world tend to grow as time goes by, since these objects take on a life of their own^10^ – particularly if they end up being widely disseminated, adopted and processed by various research groups. Again, both models and data exemplify this nicely. The very purpose of developing models is to tinker with them and modify them as required to suit researchers’ evolving understanding of the world (Morgan, 2012; O’Malley, 2008; Rheinberger, 2010; Waters, 2007) – so much so that philosophers have shifted from talk of models as objects to talk of modelling as an activity (e.g., Chang, 2012; Knuuttila, 2011). Similarly, while traditional epistemology views data as *static* products of one-off interactions between investigators and the world, the more recent relational view of data has highlighted how characteristics of these objects (such as their format, structure, order) often change when they are mobilized as evidence for claims, lending them to different types of interpretation (Leonelli, 2016, 2020). Data are mutable objects, especially when re-used within a variety of research contexts - though their characteristics can change even when they remain in the same research context, due to the evolving expectations, assumptions, technologies and materials through which they are analysed (Anorova et al., 2017; Leonelli & Tempini, 2020).

Many methods in the sciences are explicitly devised to confront the plural and unstable nature of research and its outputs. Consider bacterial kinds and related data, models and taxonomies.^11^ Bacteria reproduce by division, but may also transfer genes from one cell to another, sometimes to a very distantly related cell, a process referred to as horizontal gene transfer (HGT). Typical bacteria also mutate frequently. How do there come to be kinds with reasonably predictable properties? There are several answers. Descent from a common ancestral cell will produce similarity, though over time this will be modified by mutation and HGT, and cells to which such changes have occurred will give rise to new kinds of descendants. Among some kinds of bacteria there is a good deal of HGT, mainly limited to homologous recombination among closely related organisms, and a “species” analogous to those maintained by reproductive links in sexual species may emerge.^12^ Selective pressures may also shape organisms to a particular suite of traits that fit a particular niche.

How do scientists interact with this diversity? There are numerous approaches to bacterial taxonomy, suited to serving different ends. Data on particular genes, known to exhibit little change in sequence, are sometimes used as an empirical ground to produce taxonomies. But because of lateral gene transfer, meaning that bacterial individuals acquire their genomes from multiple ancestors, individuals can be classified very differently depending on what genes – and related data – are selected for consideration when producing a taxonomy. Hence, whether epistemic objects such as a kind name or a specific data cluster are suited to describe particular processes in nature will depend on which taxonomic decisions have been made. And the general characteristics of these epistemic objects themselves affect how taxonomies are constructed.

The mutable nature of objects and phenomena reified through research is even more evident in another type of epistemic object routinely used to study bacteria, the model organism. Much understanding of bacteria has been derived from the concerted study of specific and highly standardised bacterial strains, such as strains of *E. coli*, carried out in laboratories around the world. This approach requires researchers to devote considerable effort to keeping bacterial strains as stable and homogeneous as possible (O’Malley, 2014). This is because even under laboratory conditions strains are liable to evolve, and different instantiations of a strain may diverge, making it hard to compare results across research sites. When the strains are highly stabilised, as can be the case for model organisms and cell cultures grown in artificially produced chemical environments, it is easier to achieve and defend the validity of such comparisons (Ankeny & Leonelli, 2020; Landecker, 2007). However, inference from the properties of those standard strains to anything in nature becomes problematic as nothing in nature may correspond to the particular stable form achieved under laboratory conditions; and the stability of the standard strain is itself always open to question.

None of the above is news to microbiologists, many of whom have long recognised the implications of such reifications on their interpretations of data, taxonomies and models. However, it may be challenging to those philosophers (and even some scientists) who think that there are discrete kinds in nature, defined by essential properties and given names by their scientific discoverers.^13^ Instances of some of these kinds, they may suppose, live in the scientists’ test tubes and what is true of them by virtue of their essences must, it seems, also be true of their conspecifics in nature. The partial stabilisation of the thing in nature and in the laboratory in ways that may well not match and may, even if they once matched, cease to do so, raises concerns to which such views are oblivious. These are not merely theoretical concerns. When situations arise in which intervention is desperately needed, such as the coronavirus pandemic, scientific knowledge is regarded as the necessary ground for such intervention. But we lack an adequate philosophical account of how scientific knowledge can inform intervention in a world of constant flux.

It is as a step in the direction of such an account that we have proposed the distinction within research practice between means reification (M-Re), the initial construction of epistemic objects, such as models, data, theories or taxonomies, from phenomena, and target reification (T-Re), a specific rendition of the phenomena obtained through the investigation and manipulation of epistemic objects. The central reason for clearly distinguishing these two forms of reification is that in the real world both the epistemic objects produced through M-Re and the phenomena produced through T-Re are highly dynamic, and there is no reason to expect that the ways in which they change over time will align. Hence, if we imagine that we have a timelessly fixed representation of a static thing we are not only likely be in error, but the error is likely to increase as the epistemic object and the phenomenon follow their divergent paths.

## Reification at work: The study of viruses in biology and biomedicine

In this section, we shall illustrate how these two forms of reification have worked in biological and biomedical research on COVID-19, and thereby reflect on the dangers involved in failing to acknowledge their epistemic significance. Let us start by considering some current biological knowledge about viruses. Viruses are commonly viewed as things with a fixed chemical structure, typically a genome with a protein coat. This is actually the virion, the more or less stable part of the virus life cycle. The Sars-Covid-19 virion has a large (for a virus) RNA genome, a protein capsule, an external lipid membrane and the famous spike proteins sticking out from the membrane. An interesting point is that the membrane is composed of lipids captured from the previous host cell. This tells us first that not all virions are the same but also, more interestingly, that they carry some of their particular history with them.

The virion is the stable phase of the virus life cycle, and it is what people fear finding on door handles or floating in exhaled droplets. But the virion is not the virus, any more than the acorn is the oak.^14^ The life cycle of the virus includes a much more active phase: after the virus has inserted itself into the host cell membrane it releases its genome into the cell, and various chemical interactions occur, resulting in the release of newly formed virions and, in many cases, the lysing of the host cell. During this phase, various chemical activities are involved in the co-option of the host’s resource into the production of new virions, but none of these has any clear claim to be *the* virus. In fact, some RNA viruses may persist for long periods of time as no more than a piece of DNA attached to, or inserted into, the host genome. The upshot of all this is just that a virus is not a thing (the virion) but a process, which includes a flow of activity through a host organism (Dupré & Guttinger, 2016); or, better still, as we shall now explain, a flow through a host population.

Realisation of the fluid nature of the virus enables us to move beyond a sole concern with the “individual” virus—since there really is none—and ask about the scale at which the viral process has the effects in which we are interested. This is not a “merely philosophical” point. Crucial aspects of the behavior of the virus will be missed if one focuses solely on the capacities of the single virion. The success (from the virus’s point of view) of an interaction with a host actually turns out to depend crucially on the size of the viral interaction. One reason for this appears to be that the virus depends on the diversity of genomes within the total flow (Vignuzzi et al., 2006), and an optimal rate of evolutionary change, sufficient to defeat the immune response, but not so great as to disable the viral flow. Viral clouds, or viral quasispecies, are often the units of viral infection (Domingo et al., 2012). This is one reason why for many viruses there is a threshold dose of viral infection required to get sick, something that may seem surprising if one thinks of one virion invading a cell and allowing the cell to produce multiple virions, launching a chain reaction. But interactions between processes, not to mention those evolved in response to one another for millions of years, are often dose dependent. No one is surprised that intersection with a tsunami is a different experience from intersection with a light mist; the properties of a drop of water are insufficient to understand this kind of intersection.

Let us now relate this account of the virus life cycle to the model of processual research sketched in the preceding section, and particularly the characteristics of the epistemic object generated through M-Re. The standard molecular machine model of the virion is an epistemic object that is suited to addressing questions about the structure of the virion qua phenomenon, and that can tell us important things about the chemical resources deployed when a virion interacts with a cell. But if we are interested in the interaction of the organism with a viral flow, a different epistemic object may be needed, the viral quasispecies. And if this is the appropriate epistemic object, we immediately face the problem that it is a rapidly evolving entity. Any description of the quasispecies is expected to be rapidly out of date. Indeed, any particular description even of a virion, a “thing” that constantly emerges from the viral flow, will be one of a wide and rapidly changing repertoire of possible entities. Hence what we are learning about is not necessarily the virion or the viral flow as it exists in the world, but rather as the target conceptualised through reification.

How does the reconstrual of research from object-to-phenomenon as M-Re to T-Re affect our understanding of research findings in this case? First, it should draw our attention to the limitations of the virion-as-molecular-machine model. Such a model naturally motivates pharmacological or immunological interventions aimed at blocking the pathological interaction between virion and cell. But viral flow models tell us that this is only likely to be successful if we target a feature of this interaction sufficiently fundamental to apply to all the variants of the virus found in the quasispecies and even to all readily accessible mutations from these. As is now familiar, even when such interventions work, they may not work for very long. They are likely to fail if they do not account for the population level interaction between virions and cells, both liable to evolve new properties during the course of infection.

The processual character of an epidemic is addressed explicitly in epidemiological models, concerned with understanding the viral flow through a population. This presents quite different problems from those of intervening in the mechanistic interaction between virus and cell. Epistemic objects in epidemiology include herd immunity, the state in which the rate of transmission of a pathogen is below the rate of recovery, so that infection rates cannot increase and R, the reproduction number, or the average number of people infected by each infected person. R is of course a variable, and in addressing an epidemic the objective is to reduce it below 1 by reducing behavior liable to transfer infection. No one would interpret R, an epistemic object resulting from M-Re, as anything but a quite rarified abstraction from a set of data. And the population to which R immediately applies, the phenomenon derived from T-Re, is defined no further than a set of individuals with behaviours relevant to the transmission of viral infection. Both R and populations are well understood to be highly fluid entities, thus seemingly well-suited to capture a world in flux.

But when research moves from population dynamics to individual pathology, it tends to abandon the processual in favor of a focus on mechanical interactions between individual virion and cell, thus fostering what we regard as misguided T-Re. It is quite legitimate to take entities like virions and cells as appropriate epistemic objects for investigating the virion/cell interaction. Trouble arises when such objects are used to investigate population-level infection, however, since for reasons just explained, it is a mistake to think of the population-level infection as merely a large number of virion/cell interactions. A proper processual understanding of the population-level phenomenon requires focusing on features of the population that are invisible to the individual level reifications. For example, the mutation rate is a variable feature of the viral quasi species, and one that selection is likely to optimize for the virus, but a property that makes no sense in application to an individual virion. Yet it may well be fundamental to the dynamics of an infection event. If it is too low the virus will be unable to evade the host immune system, and if it is too high, too great a proportion of dysfunctional variants will be generated. One proposal for counteracting viral infections is to intervene to increase the mutation rate beyond this optimal point, perhaps to a point that entirely blocks the flow of viral activity (“lethal mutagenesis”; see Domingo et al., 2012).

## When is reification pernicious?^15^

The crucial issue that we wish to reiterate is that we have no reason to assume that the ways in which epistemic objects and phenomena change over time track one another. As we saw in the above case of data and models used to study viruses, the processual nature of data and models can be out of step with the processual nature of target systems. This potential mismatch is a cause of worry for many researchers, and often features at the center of scientific controversies (for instance, ongoing debates over the validity of model organisms as models, or around the reproducibility, quality and relevance of big data). However, the mismatch can be downplayed or forgotten outright; this happens most often when scientific results are lifted from their specialist domain and used as “travelling facts” elsewhere (Morgan, 2010). This is a particularly pernicious problem in big data analysis, where situated assessment of data provenance is often unfeasible or side-stepped, and data infrastructures, data mining algorithms and data models are sometimes taken to provide access to stable and reliable representations of the world whose interpretation is independent of context (Leonelli, 2020). It is also a problem when rushing to align results acquired from multiple research domains to inform specific forms of intervention, as researchers have had to do in the case of COVID – a situation exacerbated by facile linear narratives around how to translate biological knowledge into effective medical treatment.

Indeed, despite the rich biological understanding of viruses and the fast accumulation of resources to study SARS-COV-2, there are many examples of the contextuality and sensitivity of virological results being disregarded in the name of producing reliable, secure, relevant responses to the outbreak that can be tested and implemented as quickly as possible. One example is the lack of coordination around the naming of COVID variants, resulting in a proliferation of names attached to “variants of concern” (that is, variants that prove particularly infectious or deadly to humans). This situation, which the authoritative *Nature* journal declared “a bloody mess” (Callaway, 2021), was initially regarded as unimportant as researchers scrambled to acquire insight into any and all existing strains of COVID, thereby producing taxonomic epistemic objects meant to capture the characteristics of the strains being considered by each lab. But confusion began to emerge when it became necessary to compare systematically variants of key importance for public health authorities, such as the B.1.1.7 variant (also called “Kent variant” and “20I/501Y.V1”, among other names) discovered in England in 2020, which subsequently swept through Europe causing another wave of lockdowns.

The confusion around naming made it difficult for the many research teams devoted to such work around the world to discuss their findings with each other before making them public. A complicating factor was the diverse expertise of researchers and commentators involved in this work, which included taxonomists, genomicists, virologists, and experts in epidemiology, infectious diseases and public health – as well as journalists, concerned citizens and policy-makers wanting the latest updates on the evolution of the disease. Given the diverse interests, criteria and methods used by this motley crew, and the lack of norms regulating such multidisciplinary exchanges, it is no wonder that the labels attached to what was (more or less) the same object proliferated; yet this made it ever more difficult to determine whether results collected by different labs were about the same variant or not, and what future trends such results could indicate. In our terminology, this was a case of proliferating approaches to M-Re, resulting in disparate epistemic objects which were hard to relate back to their target in a consistent manner – a key problem being the difficulties in reconstructing how M-Re was established in the first place, and how different approaches to M-Re construction informed the interpretation of T-Re. To address the problem, the World Health Organisation called for the rapid adoption of a universal naming system (Nature editorial 2021) and endorsed one of the methods already in place. This solution was effective in eliminating confusion and lack of clarity around M-Re, thus providing a less ambiguous approach to T-Re. However, this solution did not pay much attention to what sources of knowledge were left behind as a result of that choice of standard, and thus to what could be learnt from other approaches to M-Re.^16^

Another instance is the calculation of infection rates from data derived from COVID testing. Calculations of infection rates depend on assumptions built into the data, such as who is being tested (asymptomatic or only symptomatic) and whether test results are automatically inserted into the counting system or not. These assumptions have changed rapidly as testing regimes have shifted multiple times both across countries and within the same country, in response to the varying resources and political priorities within each affected territory. As a result, during the first year of the pandemic the systems of inquiry used to track the spread of the virus, and related forms of M-Re and other epistemic objects, have arguably changed as fast as the virus itself – a situation that will not easily stabilize given the highly diverse and dynamic social conditions involved. And yet, the data produced on the virus and infected individuals, as well as the models used to calculate infection rates, are often treated as stable objects that can be interpreted in the same way no matter how and where they were generated. Data have been reified and treated as stable epistemic objects that can be easily assembled, modelled and compared – and with them, the phenomena that data are supposed to help to model have also been reified without critical consideration of the diverse forms of M-Re and T-Re involved in their conceptualisation. Countries have based their policies on these assumptions, and successes and failures of containment policies are being evaluated in relation to the resulting numbers, with unsurprisingly controversial and contested results.

Yet another example of problematic reification has been the rush to mine genomic and clinical data, in the hope of finding markers that increase the accuracy and speed of diagnostic tests. This process was highly successful in the short term, and yet little attention has been paid to:

1. the longer-term reusability of models and data themselves: the absence of a critical discourse on the M-Re and T-Re involved in re-using legacy biological and epidemiological data can hamper the construction of a resilient knowledge base of trustworthy and reusable findings;
2. the geographical diversity of data sources and especially findings coming from countries with less research visibility: using epistemic objects (such as infection rates) derived from very selective datasets from affluent countries, without duly considering the bias implicit in such an M-Re process, can result in inferences that are wrongly assumed to hold also for poorer regions with very different infrastructures and social conditions – in other words, in a questionable extrapolation of T-Re^17^;
3. the clinical observations by medical doctors from non-English-speaking countries, which meant overlooking sources of evidence – such as early observations of a drop in oxygen saturation levels for affected patients – that turned out to be highly significant means of diagnosis and treatment: thereby, in our terms, overlooking the availability of M-Re resources that would have meaningfully informed the subsequent pandemic response (Krige & Leonelli, 2021); and
4. the observation and experiences of COVID-19 patients and related groups, whose early involvement in the process of data collection could have helped to identify and better study the dangers presented by long-term consequences of COVID infections, thereby helping to identify “long COVID” as a phenomenon requiring urgent investigation (Leonelli, 2021).

In short, many of the data infrastructures and mining tools used to collect, model and analyse COVID-related data ended up assuming a static definition of the “knowledge base” around the disease, and made few systemic provisions for capturing changes in phenomena and responding to the temporality and specific provenance of data. This was largely the result of practical constraints such as lack of resources and time, yet it ended up affecting the quality and applicability of the resulting epistemic objects and, in turn, of the new insights produced on the phenomena of interest. As we noted in the previous section, focusing on the unique structure of a stable virion may miss the factors that determine disease severity. Evidence of just this possibility was provided in a 2020 paper by Al Khatib and co-workers, who reported that while there was a high level of similarity (99.8%) in consensus genome sequence among SARS-Cov-2 patients, finer-grained analysis revealed significant differences (Al Kathib et al., 2020). Patients with more severe symptoms displayed significantly higher within-host diversity compared to mild cases. Higher within-host diversity was also found in patients over 60, suggesting that the observed diversity was an outcome of the interaction with an older immune system rather than merely bad luck in the population with which the patient interacted.

To understand fully the ways in which the virus causes disease, it appears, it is best understood as a flow with variable characteristics, and one that is rapidly changing even within the single patient. We have already noted the difficulties in agreeing even on synchronic nomenclature for significant known variants of the virus. Here we add that the simple shifts from the reified accounts of the “original” or “Chinese” variant to the “UK variant” or “South Africa” variant provide a simplistic and potentially misleading account of the diachronic evolution of this virus, even over short periods. When such accounts are entrenched within data management systems and infrastructures, simplifications that looked useful in relation to specific short-term goals may end up scaffolding further research addressing different questions, thus restricting both researchers’ capacity to develop their thinking and the types of data identified as relevant for future research (Caporael et al., 2014).

## Studying COVID science in terms of processes, not outcomes

How does one address these issues in scientific practice? We argue that a process ontology that extends to both subjects and objects of knowledge shifts the burden of proof for researchers seeking to ground claims in empirical evidence. What needs investigation and empirical grounding are not so much the conditions under which target objects change, but rather the conditions under which such objects can be assumed to be stable, which includes the explicit consideration of both M-Re and T-Re. A properly processual epistemology would *not* assume that phenomena are persistent with the possibility of change, thus treating change as an exceptional, unusual circumstance and relying on models, data and representations of the world as an ensemble of static objects. Rather, a processual approach to knowledge production would place the idea of change as a condition of persistence at the centre of researchers’ working assumptions about their targets. This means explicitly acknowledging that no scientific method, no matter how comprehensive and dynamic in nature, can capture anything but a stabilised yet open-ended documentation of ongoing processes; and that the objects produced in the attempt to capture processes are themselves continuously modified and adapted to researchers’ changing needs and interests, in what Andy Pickering famously described as a “dance of agency”.^18^

There is no escaping the need for reification to generate knowledge. Moreover, there are no obvious general criteria to rule out specific forms of reification as intrinsically pernicious: identifying which forms of reification may be damaging is a highly situated problem, which depends on the specific conditions, goals and settings of research – including the normative commitments of researchers. A process-friendly epistemology needs to focus on the intersection between research procedures and methods (the “knowing how” required for the production of epistemic objects through M-Re) and knowledge content (the “knowing that” generated when applying epistemic objects to the study of phenomena via T-Re). Data, models, photographs, even videos capture only some characteristics of the changing world. The analysis of these research outputs needs to take this partiality – and particularly the unavoidable stability of those documents, which is central to their use as epistemic objects and yet limits their evidential and representational power – explicitly into account, and evaluate it in relation to the circumstances and goals of the inquiry at hand. By insisting on the entanglement of the researcher and the subjects of research, a processual perspective not only makes sense of the familiar ways in which science constructs its research objects, but also stresses the plastic and developing nature of researchers as biological and social beings with shifting values and normative goals – in line with much literature in science studies as well as philosophical critiques of the fact/value distinction. The many different possible ways that researchers can interact, over time, with the material entities they study makes it easy to see why this is a pluralistic view of science. At the same time, there is nothing unreal, or made up, about either the interactions or the entities interacted with: the position is equally clearly a realist one (Chang, 2012; Dupré, 2018).

The explicit adoption of a process epistemology has substantive implications for the priorities and concerns guiding everyday research practices. When operating in this way, researchers integrating data collected from different sources need to question what assumptions each source has made about the *stability* of the underlying phenomena, and evaluate whether such assumptions continue to hold and how they relate to the epistemic objects produced through M-Re. Researchers collecting data also need to prioritise methods to track and account for the spatio-temporal dimensions of research, including systems to collect accurate metadata on how, when and where data are collected as well as how data are processed in subsequent uses (Leonelli & Tempini, 2020). Unsurprisingly, new developments in data management focus precisely on the importance of collecting and linking such metadata to the data, which is widely viewed as a way to guarantee the quality and reliability of the analyses subsequently produced (Wilkinson et al., 2016). Indeed, process epistemology is well-aligned with research policies that foster critical approaches to interdisciplinarity, acknowledge the challenges associated with the wide diversity of available expertise and knowledge sources, and support dialogue and ongoing scrutiny on all aspects of the research process.

With this characterization of process epistemology in mind, let us return to the case of the COVID pandemic to reflect on (1) the opportunities created by scientific approaches that explicitly identify forms of reification and interrogate their implications for prospective uses of the knowledge being produced, and (2) the risks involved in failing to acknowledge reification and its epistemic roles in scientific inquiry. As a starting point for investigation there is nothing wrong with exploring the interaction of a virion with a human cell. We can even use a cell from a (more or less) stabilized cell line and a virion with well-established chemical characteristics. What we need to remember is that this (relatively) simple, M-reified and stabilized system expands in many directions, and each of these provides contexts that change the characteristics of the T-reified phenomena, and contexts that may themselves change in divergent ways. One of these contexts is the research system in which the interaction occurs. A second is the wider context of the virus, both as part of a recurrent life cycle (which may indeed be the immediate target of research) and as part of a population of viruses, a quasispecies, further aspects of which may affect the impact of the virus in a real life context. A third is the location of real cells in all the unimaginable complexity of the human body—organs systems, immune responses, microbial symbionts, and so on—the reactions of the virus on which may well effect one another. We are not proposing that science must somehow investigate all of these contexts simultaneously, but rather that researchers should never forget that the stability of their results is constantly threatened by the evolution of these surrounding systems. The achievement of stable, even sometimes reproducible, results is achieved by the often ingenious use of controls; but controls are, at the same time, reminders of the great difficulty of transferring our findings into the uncontrolled world beyond the lab (Güttinger, 2019). And indeed, how viruses are studied and how the resulting knowledge is collated and linked with other research results has large implications for epidemiology and public health: the reifying assumptions underpinning the epistemic objects in question need to be explicitly monitored and re-assessed as soon as those objects are put to new uses and related forms of T-Re.

We should also note how just as a wide range of social, economic and institutional factors have a profound effect on the putative stability of phenomena and epistemic objects, such factors also shape the priorities and resources used by scientists to plan and conduct their investigations.^19^ An obvious example is the extent to which the research environment in which most biologists work is shaped by reward systems and incentives focused on the quantity and short-term impact of results. In fields at the frontline of COVID-19 research such as biology, biomedicine, epidemiology and data science, scientists are primed to organise their work in “minimally publishable units” specific to their existing skills and expertise, which allow them to publish quickly in prominent academic journals. This focus distracts attention from the long-term dimensions of research and disincentivises the search for resources to develop meaningful engagement with publics beyond the narrow group of academics that may act as referees for publications. This, in turn, means that researchers have little time, funding or incentive to develop datasets and models for longer-term re-use by multiple stakeholders; to consider diverse types of data sources and how their own work relates to data obtained through different approaches, materials and research conditions; to reflect on the significance of reifying assumptions made in the lab when results are placed in a different context; or to discuss potential implications with communities that may be affected. This combination of incentives and constraints has the effect of further entrenching the reifications enacted in the course of any one research activity, making it hard to question its continuing relevance and identify related risks as results move away from their original context.

This system has generated ingrained reifying assumptions within COVID-19 research, which have made it difficult to contextualize the biology of the pathogen and its human hosts into the complex socio-political landscape of the pandemic. Such a broad contextualization has been acknowledged by many epidemiologists as essential to research on the pandemic, especially in view of the fundamental role that existing inequities in human societies are playing in determining the spread and rates of transmission. It has been suggested that the very concept of pandemic as a target phenomenon should be framed to acknowledge the significance of social factors, for instance by shifting to the idea of “syndemic”, defined as “a set of closely intertwined and mutually enhancing health problems that significantly affect the overall health status of a population within the context of a perpetuating configuration of noxious social conditions” (Bambra et al., 2020, 13). In fact, the interdependence of biological and social factors, and the role this plays in evaluating the relevance and implications of using certain epistemic objects and focusing on specific phenomena, has long been highlighted in public health and disaster studies (Louissaint, 2021). What makes it so hard to consider within biological research is, we argue, the way in which reifying assumptions become invisible scaffolds within research systems, uncritically used to support continuing use of available epistemic objects and to entrench specific interpretations of their outputs (Caporael et al., 2014).

## Conclusion: Process epistemology and conceptions of knowledge

We conclude with a reflection on the value of process epistemology as a corrective to essentialising views on how empirical knowledge is produced, views which fail to stress the epistemic importance of remaining alert to reification and its consequences; and we draw out some implications of this analysis for our understanding of the nature of knowledge.

Consider a statement such as the genome sequence of Sars-Covid-2 is X. How might this be considered true and justified? A standard view of its truth would be that this sequence is a property of this virus. And yet, the sequence is an epistemic object and the virus is a phenomenon. The standard account thus constitutes a kind of category mistake: epistemic objects are not properties of phenomena. But more important to our current purposes, the statement freezes the relationship, whereas we know that it is an abstraction (few or no actual virions have exactly this genome sequence) and that its relation to phenomena is constantly changing (the distribution of genomes is evolving over time). How is such a statement justified? By some interaction between a set of genomes and the sequencing machinery in a respectable lab. When attempting to acquire insights on the properties of a given strain of Sars-Covid-2, it may be perfectly acceptable to rely on M-Re to produce sequence data and T-Re to infer insights about that strain from an analysis of such data. But recognising the reification processes involved in such research, and the limits and constraints attached to such processes, may help prevent problematic inferences such as the use of those same sequence data to produce knowledge about all Sars-Covid-2 genomes. Using data obtained from a single strain to generate knowledge about all existing strains constitutes an unjustified, even dangerous reification. What we need to know in order to use data properly as epistemic objects is where and when those objects were produced, with what equipment and methods, and with reference to which sample. In other words, we need to know the circumstances and methods underpinning M-Re. This gives us at least a chance of assessing what the resulting epistemic objects might appropriately be used for – what forms of T-Re are best associated with the outputs of this M-Re.

Rather than “the genome sequence of Sars-Covid-2 is X” being a paradigm of knowledge, we might thus focus on a statement such as “this test provides the capacity to identify Sars-Covid-2 infections that include this genome sequence”. The test is here conceptualised in a way that can be passed on to other researchers and which needs to be performed in specific ways in order to generate meaning. In this sense, what researchers can do (and by implication cannot do) with the test and its results can be clearly stated on the tin. The implications of this account for existing philosophical theories of knowledge are beautifully captured in this remark by Chang (2017): *“knowledge-as-information may only be flickering moments in the continual creation and use of knowledge-as-ability, and propositional belief only occasional crystallisations in that flow of activity.”* We agree that process epistemology views knowledge as ability as prior to – and enabling the existence, understanding and use of - knowledge as information.^20^

This brings us back to where we started, that is with the famous Putnam/Kripke account of natural kind terms. Nothing could be more disastrous to the scientific research we have been considering than the idea that the word “virus” was attached by a “dubbing” event, perhaps when Pasteur referred to the agent responsible for rabies, to some essentially defined natural kind of phenomenon. The rabies virus is not merely very different from the Sars-Covid-2 virus, but the rabies virus in France in the 1880s was probably significantly different from that present today in India. And even the RNA strands in a particular rabies infection are likely to be importantly different. Much better to adopt a Wittgensteinian focus on the uses of a word such as virus, and to reflect on the conditions under which such uses prove most fruitful. As we have shown these are many and diverse. The curation and coordination of such uses is an essential aspect of an effective scientific method.

## Footnotes

1 Throughout this paper we shall refer to entities in the world, the targets of scientific investigation, as *phenomena*, and we shall use the term *object* to refer to the entities that science constructs to further this investigation (concepts, models, data, theories, etc.). For a congenial use of the term phenomena, see Massimi (2022).

2 Note that stabilization is a necessary feature of reification in our view, but it is not synonymous with it. Rather, reification is a very specialized form of stabilization that takes place within the process of inquiry, and is therefore part and parcel of any attempt to obtain scientific knowledge.

3 While there are obvious connections with such earlier uses of this word, most notably by Marx, our usage here is not intended to be identical to any of these. The closest historical usage within philosophy is that of William James and John Dewey, who also place this notion at the centre of their versions of pragmatism (e.g. Dewey, 1925; James, 1907). Winther (2014) provides a detailed analysis of James’ and Dewey’s treatment of reification as a “pathology of abstraction”.

4 Several philosophers have objected to this claim on the grounds that Aristotle, the pre-eminent theorist of substance, understood substances in a way compatible with the features of life that point towards a process ontology (Steward, 2020; Austin, 2020; Oderberg, 2020; Wiggins, 2020.) This is not the place to respond to these objections, which we do not see as critical to our present argument. To sidestep this problem, in this paper we shall generally speak of things rather than substances. However, we must note that our use of “thing” is more restrictive than the everyday use of this extremely general term. We hope our usage will be clear enough in context.

5 For much more detailed argument, see Dupré and Nicholson (2018) and various other papers in Nicholson and Dupré (2018).

6 It is often erroneously supposed that the genome, or genome sequence, provides such essential properties. For an explanation of the fluid and diverse character of the genome, see Dupré (2010).

7 By “capturing” aspects of the world we mean referring to such aspects in ways that make it possible to investigate them. Capturing can thus take the form of “documenting”, “representing” or “imagining/idealizing” aspects of the world, depending on the situation. While philosophical debate continues over the accuracy and realism with which models, theories and data represent, abstract and/or idealise reality, we remain agnostic on this issue within this paper; all that matters for our purposes is that researchers intend to use a given dataset/model/theory to “stand in” for the feature of the world they are interested in, and aim to extract knowledge about the world from that object.

8 We are indebted to Hans-Jörg Rheinberger for the idea of epistemic object, though our use purposefully embraces both what he calls “epistemic things”, the material objects that constitute the object of inquiry, and at least some of what he calls “technical objects”, i.e. the experimental tools through which inquiry is performed (1997, 27–31). This is because for the purpose of our analysis, the relevant distinction is between phenomena in the world and what researchers use to investigate those phenomena, which encompasses both the objects taken to represent phenomena and the objects used as instruments for investigation. Our notion of epistemic object thus embraces any object produced for the purpose of scientific investigation, no matter how critical that object is to a specific research tradition or epistemic community, and including – but not limited to - experimental practice. As Rheinberger also notes, the designation of such objects as “epistemic” involves some degree of social consensus among scientists – so that a model or datum recognised as such by a single scientist is not really an epistemic object (and indeed not even a bona fide model/datum, as argued in Leonelli, 2016, ch.3).

9 See for instance Wimsatt (2007), Morgan (2012) and Frigg and Hartmann (2020) among many others.

10 When saying that epistemic objects take on “a life of their own” we do not mean that they literally become alive, but rather that their material characteristics shift in response to changes in their surroundings (including how they are handled by human agents).

11 For more details on microbial research practices, see O’Malley (2014).

12 Bobay and Ochman (2017) argue that the substantial majority of bacteria belong to such biological species.

13 Contemporary essentialists include Devitt (2008, 2010) and, from a more purely metaphysical perspective, Oderberg (2007). It is harder to find scientists explicitly endorsing or rejecting essentialism, but essentialist assumptions are sometimes implicit in scientific work.

14 Whether a virus is an organism, or even alive, remain much debated questions. For various perspectives, see Villarreal (2004), Van Regenmortel (2016), Dupré and Güttinger (2016).

15 Winther (2014, 2020) uses the expression “pernicious reification” in a sense closely related to what we discuss in this section. Winther’s category is broader than ours, applying generally to inappropriately literal interpretations of models.

16 For example, currently standard classification of Covid variants is based on lineages with features of interest. But other significant genes can differ within a lineage, and the same mutation can give rise to distinct lineages with properties differing on account of other aspects of the genetic context. Perhaps more significantly, if indeed the distribution of phenotypes within a viral swarm is a determinant of the virulence of an infection, then different viral flows within a lineage may have significantly different pathological properties.

17 Even initiatives specifically set up to promote collaboration and wide-ranging data sharing with low-resourced research environments and researchers from the Global South, such as GISAID (https://www.gisaid.org/), were attacked as obstacles to the quick dissemination and interpretation of data (van Noorden, 2021).

18 Epistemic objects such as data, models and so forth are thus not merely “interpreted”, but need to be performed in specific ways in order to acquire meaning (Pickering, 1995).

19 A comprehensive discussion of these social dimensions of research is beyond the scope of this article, but we would like to signal the relevance of the rich and growing literature on reward systems within the philosophy, history and social studies of science.

20 There are clear parallels here with Ian Hacking’s emphasis on the importance of intervention and his later “dynamic nominalism”; and with the view of scientific understanding as grounded in the capacities, skills and commitments of those using research outputs (as defended in de Regt, Leonelli and Eigner, 2009).
